# An Investigation into the Sensory Properties of Luffa (*Luffa cylindrica* (L.)) Fruit Powder

**DOI:** 10.3390/foods14152594

**Published:** 2025-07-24

**Authors:** Matthew Code, Matthew B. McSweeney

**Affiliations:** School of Nutrition and Dietetics, Acadia University, Wolfville, NS B4P 2R6, Canada; 0304998c@acadiau.ca

**Keywords:** luffa, novel ingredients, sensory perception, sponge gourd, check-all-that-apply, consumer perception, hedonic, food neophobia, new ingredients

## Abstract

Luffa fruit is an underutilized and novel ingredient in North America. To increase the shelf life of luffa fruit, this study evaluated the creation of luffa fruit powder using three different drying temperatures (40 °C, 50 °C, and 60 °C). The objective of this study was to evaluate the sensory properties and acceptability of luffa fruit powder with unfamiliar consumers (those who do not regularly eat luffa fruit). Participants (n = 88) evaluated the luffa fruit powders mixed into couscous, as well as a control (couscous without luffa fruit powder) using check-all-that-apply and hedonic scales. Furthermore, the participants were asked how they felt about luffa fruit powder after evaluating the samples (comment question). The hedonic scores were not significantly affected by the addition of the luffa seed powders dried at different temperatures. However, the luffa seed powder was associated with a mild flavour, as well as being described as earthy and vegetal. The participants did indicate that the luffa seed powder added moistness to the couscous. As the drying temperature increased so did the intensity of the flavour. Furthermore, participants indicated they would be interested in luffa seed powder if it has nutritional benefits. Overall, this study investigated the sensory properties of a novel ingredient, luffa seed powder, and future studies should continue to explore its sensory properties and chemical components.

## 1. Introduction

*Luffa cylindrica* is a member of the cucurbitaceous family—commonly called a sponge gourd, loofa, vegetable sponge, or bath sponge [1]. Other members of the Cucurbitaceae family include gourds, pumpkins, cucumbers, and zucchinis. The sponge gourd is cultivated for a wide range of uses. In North America, the sponge gourd is most commonly used for its exfoliating properties; but, *Luffa cylindrica* has been utilized as a staple ingredient in many cuisines worldwide [1] and has a role in traditional medicine [2]. The fruit in the sponge gourd must be consumed when the fruit is immature. As the gourd matures, it produces a fibrous matrix that is desirable as an exfoliator but lacks acceptable sensory properties.

Consumers continue to demand foods that are functionally beneficial and healthy, especially as the rates of chronic conditions continue to rise [3]. Consumers are expecting more from their foods and looking to their diet to help support healthy living. The market of nutraceuticals and supplements has continued to grow over the last decade and, in 2019, it was reported to be worth almost USD 353 billion [4]. Consumers are more likely to consume and be accepting of a novel food or ingredient if they have knowledge of the its health benefits. The higher nutritional awareness of a functional ingredient, the more likely it is to be accepted [5]. However, it should be noted that in the quickly evolving food industry, the number of products that have entered the market over the last decade has increased dramatically. Research suggests that new products have a failure rate of approximately 70–80% [6].

There is an opportunity for luffa fruit to be a viable source of nutrients due to its high nutritional content. In Western society, luffa is traditionally grown for the fibrous seed pod found in the mature luffa. However, in other parts of the world, luffa is a culinary staple. Cultivated in China, India, and Southeast Asia, the immature luffa fruit has been compared to that of the zucchini plant as it has a mild and slightly sweet flavour, as well as a juicy and tender texture [7]. In Asian cuisine, it is known for its ability to absorb and retain liquid, similar to that of tofu [8]. Immature luffa fruit is a good source of carbohydrates, vitamins, and various minerals [9]. In particular, sponge gourds contain multiple nutrients such as alkaloids, flavonoids, sterols, glycosides, and glycoproteins [10]. Furthermore, luffa fruit contains many saponins, glycosides, and flavonoids [11].

Immature luffa fruit is difficult to store over long periods, due to its relatively short shelf-life and difficult storage conditions. Luffa fruit can be stored for 7–21 days and needs to be stored between 10–21 °C; temperatures below 10 °C can result in a chilling injury in the immature luffa fruit [12]. Dehydrating vegetables is an old practice and may be a viable solution for extending the shelf life of luffa. Hot-air drying has been used to remove moisture and prevent the spoilage (i.e., inhibit microbial growth and slow enzymatic activity) of many fruits and vegetables [13]. Hot-air drying can also lead to new sensory properties (e.g., appearance, flavour, texture) in the food [14] and allows for a product to be sold year-round. As such, this study wanted to investigate how drying of the luffa fruit impacts its sensory properties and acceptability.

The sensory properties of a food product directly impact consumer acceptability of a food [15]. Sensory evaluation is an important step in the process of developing new foods or refining processing steps for new ingredients. This study utilized hedonic scales and check-all-that-apply (CATA) to evaluate the consumer acceptability and sensory perception of the novel ingredient, luffa fruit powder. Hedonic scales have been used to evaluate many different novel products [16,17], as well as other products incorporating ingredients from the luffa plant [18,19]. Furthermore, many studies have used CATA to evaluate novel ingredients as it allows researchers to determine which sensory properties impact the consumers’ hedonic scores [20]. CATA is easily understood by untrained participants or consumers and is able to create a description of novel ingredients and food items [21,22]. It is also less time-consuming than trained panels and can offer direction to food product developers when paired with hedonic scales based on which sensory properties positively and negatively impact consumer liking [23].

Therefore, this research aimed to explore the consumer perception of luffa fruit powder and identify how unfamiliar consumers (those who do not regularly eat luffa fruit) evaluate this novel ingredient. Furthermore, the effects of various hot-air-drying temperatures on the sensory characteristics of luffa incorporated into couscous was evaluated. Couscous has been used in past studies as a carrier for other novel ingredients [24,25].

## 2. Materials and Methods

### 2.1. Samples

Luffa fruit was provided by Annapolis Valley Luffa (Avonport, Nova Scotia, Canada). The luffa fruit was sliced to a uniform thickness and then dried for 24 h at three different temperatures (40 °C, 50 °C, and 60 °C) [26]. Luffa fruit has been described as similar to cucumber in structure and therefore the temperatures used in this study were similar to those used in a study by Guiné et al. [26] to dry cucumbers. The luffa fruit was then ground and passed through a 60 mesh sieve [27]. The luffa fruit powder was stored in a freezer (−18 °C) until the sensory trial and chemical characterization.

The proximate composition (ash [AOAC 923.03], protein [AOAC 950.36], moisture [AOAC 930.15], fat [AOAC 935.38], fibre [AOAC 991.43]) of the luffa fruit powders was determined in triplicate. The total phenolic content (TPC) of each powder was determined by the Acadia Laboratory for Agri-Food and Beverage (Wolfville, NS, Canada) in triplicate. The TPC used the Folin–Ciocalteu assay as described by Dubey et al. [28].

The luffa fruit powders were mixed into couscous for the sensory analysis trial at a level of 6% wt/wt based on a previous study evaluating the addition of a novel ingredient to couscous [25]. Couscous was chosen for this study as luffa fruit is usually used in savoury applications [7]. The samples will be referred to as 40C (couscous with the powder dried at 40 °C), 50C (couscous with the powder dried at 50 °C), 60C (couscous with the powder dried at 60 °C), and control (did not include luffa fruit powder). Couscous (durum wheat semolina; Loblaws Inc., Toronto, ON, Canada) was purchased from grocery stores and the batch numbers were matched for consistency. The couscous was mixed with the luffa seed powders (57.09 g) and then added to boiling water (156 g). The couscous was agitated with a fork and then left to sit for 10 min. After ten minutes, the couscous was fluffed with a fork. A total of 100 g portions of the couscous were then mixed with 13.5 g of olive oil and 2.5 g of salt and fluffed again with a fork [29].

The samples (approximately 20 g) were placed in transparent plastic cups labelled with random three-digit codes. The samples were served at room temperature. The sample presentation followed the design outlined in Macfie et al. [30]. There was a thirty-second break in between samples where participants were instructed to drink distilled water to cleanse their palate.

### 2.2. Participants

Participants (untrained panelists) were recruited based on regular couscous consumption (at least monthly) and if they did not have any sensitivities or allergies to any of the ingredients. In total, 88 participants (aged 18 to 30—30, aged 31 to 40—26, aged 41 to 50—16, aged 51 to 65—16; 48 females, 38 males, 2 others) were included in the sensory trial. All participants gave informed consent before participating in the sensory trial. The study was approved by the Acadia University Research Ethics Board (#13-72) and was conducted in accordance with the Declaration of Helsinki.

### 2.3. Sensory Procedure

The participants evaluated their liking of the appearance, aroma, taste, texture, and overall liking on a nine-point hedonic scale (1 = Dislike Extremely, 5 = Neither Like nor Dislike, 9 = Like Extremely). The participants then evaluated their sensory perception of the samples using CATA (the attributes included soft, hard, gritty, moist, dry, sour, mild flavour, strong flavour, savoury, aftertaste, woody, earthy, nutty, bitter, sweet, metallic, compact grains, homogenous grains, floral, citrus, off-flavour, salty, vegetal, and crumbly). The attributes were randomized [31] and were included based on a literature review [24,25,29,32,33,34,35]. The attributes were reviewed by research associates employed in the sensory lab for suitability to describe the samples. The participants were also asked if they perceived any additional attributes in the samples using a comment question. Once, they had finished evaluating the samples, the participants were asked what they thought of the luffa fruit powder. They also completed the Food Neophobia Scale (FNS; [36]) and demographic questions.

Participants completed their evaluations seated in sensory booths with questionnaires presented using Compusense (Version 25.0.32593, Compusense Inc., Guelph, ON, Canada) on iPads.

### 2.4. Statistical Analysis

The chemical composition values were analyzed using an ANOVA and Tukey’s HSD test to evaluate if significant differences existed. An ANOVA and Tukey’s HSD test was also used to evaluate if significant differences existed for the samples’ hedonic scores. The CATA results were analyzed following the methodology outlined by Vidal et al. [37]. A frequency table was created, tabulating when each sensory attribute was used to describe each sample, and then a correspondence analysis (with chi-square distances) was conducted on the frequency table. A Cochran’s Q test was also used to evaluate if significant differences existed between the sensory attributes of the different samples. A penalty lift analysis compared the relationship between the sensory attributes and the overall liking scores [38]. Responses to the comment question were evaluated using the Compusense software (Version 25.0.32593), and the authors identified recurring concepts in the responses following the procedure by Fonseca et al. [39]. The participants’ FNS scores were calculated as the sum of the scores (after the negative terms had been reversed). Scores ranged from 10 to 70 [36,40]. A Pearson’s correlation coefficient determined the relationship between overall liking the participants’ food neophobia. All analyses were completed using XLSTAT (Lumivero, Denver, CO, USA) in Microsoft Excel.

## 3. Results

The different drying temperatures did not significantly impact the protein, lipid, ash, or total dietary fibre of the luffa fruit powders (*p* > 0.05; outlined in Table 1). The drying temperature significantly impacted the final moisture content, with the 40C sample having a significantly higher moisture content than the other powders (*p* < 0.05). The TPC significantly increased as the drying temperature increased, and the 60C sample had the highest TPC (*p* < 0.05).

Mean liking scores are presented in Table 2. The addition of the luffa fruit powder resulted in a decrease in the liking of appearance in all the luffa-containing samples (40C, 50C, 60C; “Neither Like nor Dislike”; *p* < 0.05) compared to the control sample (“Slightly Like”; *p* < 0.05). There was no significant difference observed in the liking of appearance between the three luffa powder samples. No significant differences were observed in the liking of aroma (“Slightly Dislike”; *p* < 0.05), taste (“Neither Like nor Dislike”; *p* < 0.05), texture (“Slightly Like”; *p* < 0.05), and overall liking (“Neither Like nor Dislike”; *p* < 0.05) between the four samples.

**Figure 1:**
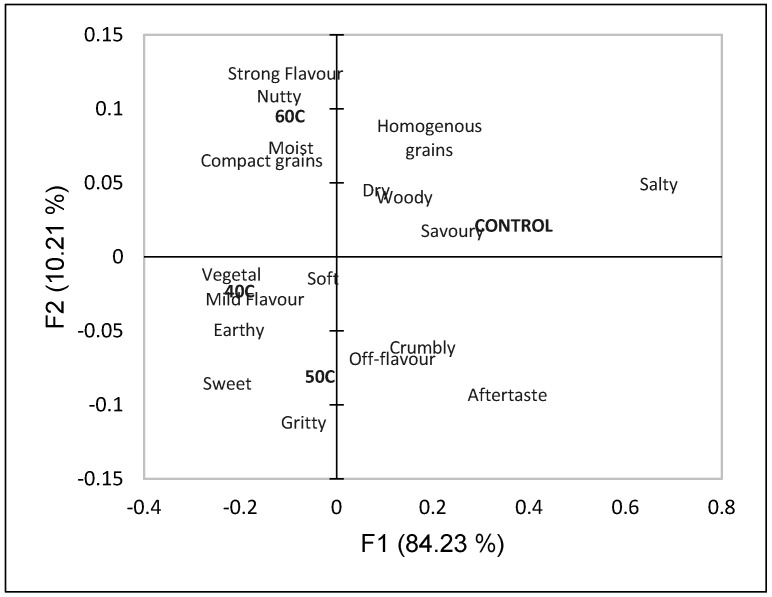
Biplot based on the first two dimensions of the correspondence analysis for the samples and the sensory properties included in the CATA question.

The participants identified their sensory perception using a check-all-that-apply (CATA) question (Figure 1). The first two dimensions of the correspondence analysis explained 93.78% of the variation (84.40% on the first dimension and 8.98% on the second dimension). The control was separated from the other samples, 40C, 50C, and 60C, by the first dimension. The 40C, 50C, and 60C samples were separated from each other by the second dimension. The 40C sample was associated with mild flavour, soft, and vegetal. The 50C sample was associated with gritty, off-flavour, sweet, and earthy. The 60C sample was associated with moist, strong flavour, and compact grains. The control was associated with salty, dry, woody, homogenous grains, and savoury.

**Figure 2:**
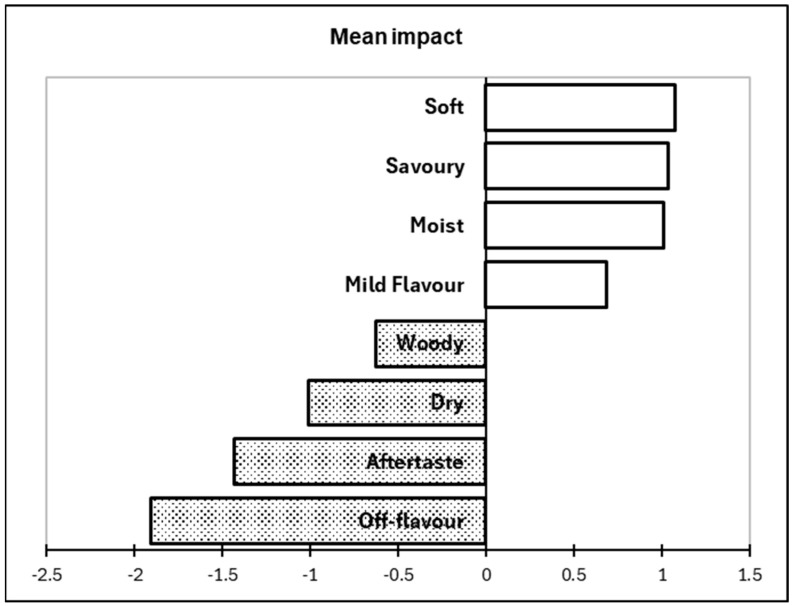
Penalty lift analysis based on the sensory terms included in the CATA question and overall liking scores recorded on the nine-point hedonic scales. The filled bars (with dots) represent attributes with a negative impact on the overall liking, and the unfilled bars represent attributes with a positive impact on the overall liking. Only the attributes that had a significant impact on liking were presented.

A penalty lift analysis was conducted utilizing the overall liking scores in conjunction with the sensory properties recorded from the CATA question (Figure 2). Attributes that had the largest positive impact on consumer liking were soft, savoury, moist, and mild flavour. The sensory properties that negatively impacted overall liking included woody, dry, aftertaste, and off-flavour.

After evaluating all the samples, the participants were asked their opinion of the luffa fruit powder (outlined in Table 3). In agreement with the CATA question, the participants identified in the comment question (Table 3) that the luffa seed powder added moistness to the couscous and had a mild flavour. Some participants identified that the luffa seed powder led to off-flavours in the couscous and off-aromas. The participants also described the luffa fruit powder as being earthy. Participants identified they would be interested in luffa seed powder if it had nutritional benefits. Overall, the participants seemed to be split on the acceptability of luffa seed powder, as many stated very positive opinions, but others were not interested in eating it again or buying it (negative).

The participants’ average FNS score was 34.1, indicating the sample population had a low food neophobia. The FNS scores were compared to the overall liking scores; however, there was no correlation between the two measurements (r = 0.145).

## 4. Discussion

Drying vegetables for storage is an old practice dating back to the 18th century [41]. Drying might be a possible long-term storage solution for the *Luffa cylindrica* fruit. The higher drying temperature increased the phenolic content of the luffa fruit powder, agreeing with past studies [42,43]. The increased amount of phenolics in the powders dried at higher temperatures is due to some of the phenolic compounds being liberated from the food matrix as hot-air drying breaks down the cellular constituents and releases bound phenolics. Furthermore, the higher temperature is able is able to inactivate or slow the activity of phenolic oxidizing enzymes [44]. The other tested chemical components were not impacted by the drying temperature.

The main objective of this study was to explore the consumer acceptability of the different drying temperatures of *Luffa cylindrica* fruit, ground into a powder, and added to couscous. First, the participants evaluated the four samples for their liking of aroma, taste, texture, and overall liking. The results illustrated that the differing hot-air drying temperatures did not have an impact on the aroma, taste, texture, or overall liking of the product (see Table 1). This was a promising finding as a practical application since it might suggest that luffa fruit powder could be incorporated into different foods without impacting consumer acceptability. Luffa fruit powder might be a valuable ingredient to increase dietary fibre intake in durum wheat applications; many studies have focused on the incorporation of functional ingredients into durum wheat pasta [45].

Luffa fruit has a mild and slightly sweet flavour [7] and the drying treatment did not drastically impact the flavour of the luffa fruit. Participants did not find any significant differences in their liking of the aroma (*p* > 0.05); this could be explained by the inherent blandness of luffa fruit. The participants selected “Mild Flavour” in the CATA question more frequently than “Strong Flavour”, with 179 responses for the former (65 for 40C, 60 for 50C, and 54 for 60C) and 53 responses (14 for 40C, 19 for 50C, and 20 for 60C) for the latter (a total for all three samples containing luffa fruit powder). Furthermore, a past study utilized experienced participants to determine attributes associated with luffa sap and identified it had a mild flavour [18]. This result agreed with a past study that used hot-air drying treatment on cucumbers (a similar fruit) and identified that it led to a mild flavour in the cucumber powder [26]. The open-ended comment question revealed that some participants thought that the samples appeared to have distinct aromas (n = 14). Examples of these distinct aromas included the control being described as smelling like playdough, cardboard, or old grains; the 40C sample was described as acceptable by some (n = 14), but disliked by others, with some participants noting that the smell was too strong (n = 8); the 50C sample had more negative comments (n = 18), with descriptors like “off-putting”, “fishy”, and “woody”, while other participants described the smell as mild or pleasant; and the 60C sample was described by some as “off-putting” (n = 10). This result agreed with a past study which identified that hot-air drying could lead to off-flavours in pumpkin powder [46], a fruit that is similar to luffa. Participants may have described the luffa fruit powder samples as off-putting due to a lack of familiarity with luffa, as unfamiliarity with a food can lead to negative feelings toward the product [47]. Some of the participants (n = 12) had strong opinions about the aroma, describing it as something that belonged in a barn and should be animal feed, yet other participants noted a smell but did not find it unpleasant (n = 10). Future studies should consider including these identified attributes, especially if they are using CATA, to evaluate products containing luffa fruit powder. As drying temperature increased, the hedonic scores for aroma liking decreased (but not significantly). Overall low scores for aroma might be explained by the fact that couscous is typically served as part of a dish rather than by itself. There was also the possibility that there was not enough luffa fruit powder in the samples to notice a difference, which may be why there were not any significant differences in the liking scores for aroma. Future studies should evaluate the sensory differences for the incorporation of increasing amounts of luffa fruit powder.

No significant differences were found in the hedonic scores for taste (*p* > 0.05). However, when exploring the comment question, varying responses were observed. The 40C sample was described as subtle, with little taste and satisfactory (n = 10). A common trend was that participants were anticipating a strong flavour due to the aroma, but most reported that the flavour was quite mild compared to their expectations. During the CATA question, there was a significant difference in saltiness between samples. Salty was selected for the control significantly more than the luffa-containing samples. An explanation for the decreased saltiness perception in the luffa fruit powder samples might be that the inclusion of luffa fruit led to different flavours that masked the perception of saltiness [48].

Significant differences in the liking of the texture were not observed in the samples (*p* > 0.05). Previous studies have reported that stickiness and mouthfeel are among the most important textural properties of couscous [49]. The textural properties included in the CATA questionnaire, in the order of most selected to least selected (for all samples), were the following: soft (200), crumbly (147), moist (128), dry (93), compact grains (79), homogenous grains (63), and hard (11). Previous studies have reported that quality couscous is associated with a soft, non-sticky granular product [25]. The 60C sample was associated with moistness and compact grains; a previous study exploring the addition of novel ingredients in couscous found that moistness increased consumer liking [50]. However, the 50C sample was associated with grittiness, an attribute that has been found to decrease the liking of couscous [50]. The 40C sample was associated with soft, which increased liking significantly in this study (Figure 2). The liking scores for texture may not have been significantly different but the drying temperature did seem to impact the textural attributes. Future studies should continue to investigate the textural properties of luffa fruit powder.

The addition of luffa fruit powder did negatively impact liking scores for appearance (*p* < 0.05). Previous studies utilizing couscous as a vehicle for the addition of novel ingredients found that the addition of novel ingredients positively impacted appearance liking scores [32], which contradicted the results of this study. This result may be explained by the appearance of the luffa fruit powder after rehydration; the 40C sample appeared grey after rehydration whereas the 50C and 60C samples appeared grey/brown. Consumers are greatly impacted by off-colours present in food, which might explain why liking scores for appearance were decreased [50]. Notably, one participant wrote in the open-ended comment question that the 40C sample appeared to contain worms. Another participant wrote that the 50C sample appeared to be mouldy. These feelings likely impacted the liking scores of appearances, based on the strong avoidance responses induced by the food’s colour [50]. Benayad et al. [33] examined an enriched wheat couscous and identified that the addition of different colours to couscous based on ingredient addition increased consumers’ acceptability; it is apparent that colour impacts consumers’ attitudes towards appearance.

The lack of differences found in the overall liking, aroma, taste, and texture could be explained by the lack of flavour of the luffa fruit powder [18], and therefore, it could be added to a food product to enhance its nutritional content without negatively impacting its consumer acceptance. Furthermore, it could be that there was not enough luffa fruit powder incorporated in the couscous to detect a difference. The amount of luffa fruit powder incorporated was chosen based on past studies that have added a novel ingredient to couscous [25]. This result agreed with a past study that identified bitter gourd (*Lagenaria siceraria*) powder, a fruit similar to luffa, did not impact the acceptability of an instant dessert mix when added at a 20% level [51]. However, it was being added as a fat replacer, which was not the case in this study. Future studies should explore increasing the quantities of luffa fruit powder to determine the optimal level of incorporation. The liking scores across all attributes including overall liking, were low (ranging from dislike slightly to like slightly). These low liking scores may be attributed to the evaluation of couscous without other foods, as couscous is normally served with other products. Future studies should look at the incorporation of luffa fruit powder into dishes that contain other ingredients such as vegetables and protein sources.

To further categorize luffa fruit powder, the sensory attributes were explored using CATA and the first two dimensions of the correspondence analysis, which can be seen in Figure 2. The first dimension explained 84.40% of the variability, with 8.98% of the variability on the second dimension for a total of 93.78% of the variance explained. The participants separated the control sample from the luffa-containing samples by the first dimension. The control was associated with savoury, salty, woody, dry, aftertaste, and homogenous grains. The participants separated the luffa-containing samples by the second dimension, with the 40C sample associated with mild flavour, vegetal, soft, and earthy; the 50C sample was associated with gritty, off-flavour, sweet, and crumbly; and the 60C sample was associated with moist, compact grains, nutty, and strong flavour. The higher drying temperature (60C sample) was associated with strong flavour (while the 40C sample was associated with mild flavour) and this may be attributed to a higher TPC of the 60C sample as liberated phenolic compounds can lead to unique and strong flavours. To further understand the characterization of attributes from the CATA question, a Cochran’s Q test was performed for each attribute; significant differences were found for earthy and salty. Multiple pairwise comparisons using the McNemar (Bonferroni) procedure determined that the control was significantly saltier than the luffa-containing samples. The difference in saltiness might be explained by the presence of other sensory characteristics, as the intensity of some basic tastes can be diminished in the presence of other flavours [48]. The control was significantly less earthy than the 40C and 50C samples, and the 60C sample was not significantly different from the control or the 40C and 50C samples.

The penalty lift analysis identified soft, savoury, moist, and mild flavours that drove the participants’ liking of the couscous. Soft was associated with all samples, with previous studies have reported softness as a key attribute to increasing the consumer liking of couscous [25]; therefore, it made sense that it had the greatest mean impact on liking. Savoury was associated with the control, whereas moist was associated with the 60C sample. Mild flavour was associated with the 40C sample. The penalty lift analysis also identified woody, dry, aftertaste, and off-flavour as characteristics that drove disliking of the couscous. Woody, aftertaste, and dry were associated with the control, whereas off-flavour was associated with the 50C sample. A study investigating the sensory properties of quinoa (a similar product) reported that woody was negatively correlated with consumer acceptance [52]. Based on the results, the 40C and 60C samples were associated with the sensory attributes that increased consumer liking, while the 50C sample was not. Although there was not a significant difference in liking, the 40C and 60C samples may have possessed more desirable sensory properties.

After evaluating the samples, the participants were asked about their overall perception of the luffa seed powder. Their responses agreed with the participants’ sensory perception (CATA question) as they identified the luffa seed powder had a mild flavour but also imparted other off-flavours and off-aromas. Luffa fruit has been described as having a mild flavour [7]. The earthy flavour agreed with a past study which identified that sponge gourd has a rich musky Basmati-like aroma that can be described as earthy [53]. Furthermore, the off-flavour may have been due to the presence of 2-acetyl-1-pyrroline (2-AP) in the sponge gourd, as it has been found in cucumber and is described as a musky aroma [54]. Previous studies have identified the presence of 2-AP in sponge gourds and ridge gourds [53,55]. The 2-AP compound can be a desirable aroma in some products (rice, cucumbers) [56], but it could be off-putting to consumers if they are not familiar with this flavour or not expecting this flavour. As stated above, none of the participants were regular consumers of the luffa fruit so their perception of this earthy flavour needs to be further explored in future studies. Participants also wondered if the luffa fruit powder was healthy for them, which reinforced that consumers are interested in novel ingredients that have nutritional benefits [57]. The last two categories were the positive and negative comments. The positive comments may have been due to the participants having low food neophobia and that they were interested in trying new food ingredients. However, the FNS scores were not significantly correlated to the overall liking scores. As stated above, consumers seemed to be interested in new ingredients that have nutritional benefits. The negative comments may have been due to a lack of familiarity with luffa fruit, as familiarity has been found to influence the consumer acceptance of food [58] and luffa fruit is not a common food in Atlantic Canada (where this study took place). The negative comments could also be due to its addition to couscous, as some participants identified they felt it would be better served as a powder added to a smoothie or in baked goods.

This is the first study that the authors know of investigating the sensory properties of luffa fruit powder, especially with those living in North America. Future studies should continue to investigate different processing techniques for luffa fruit to extend its shelf life. Future studies could also investigate different heat treatments and their impact on the resulting sensory properties, as well as the chemical composition. Studies should also investigate the volatile composition of the luffa fruit powder, as well as evaluate other chemical components of the luffa fruit powder as this study focused on consumer perception. As participants are interested in whether luffa fruit powder has nutritional benefits, studies should be conducted to investigate if there are nutritional benefits to luffa fruit powder, especially as luffa is used in traditional medicine [58,59]. Also, based on the comment questions, the colour of the luffa fruit powder should be further investigated. Firstly, the impact of the colour of the luffa fruit powder on consumer acceptability should be quantified. Secondly, the impact of the drying treatment on the colour of the luffa fruit powder should be assessed (e.g., colourimeter). A limitation of this study was that the participants had low food neophobia and this may have been due to the ethical requirement that all ingredients must be presented to the participants. The participants knew they were being asked to consume an unfamiliar ingredient (luffa fruit powder), and this could have led to only consumers who are interested in novel ingredients participating in the study. Furthermore, future studies should move beyond quantifying the TPC and identify which specific phenolics are present in luffa fruit powder and how they impact the flavour of the luffa fruit powder. Lastly, future studies investigating luffa fruit powder using the CATA methodology should expand the CATA attributes list based on the responses to the comment question in this study.

## 5. Conclusions

This study evaluated the sensory properties of a novel ingredient, luffa fruit powder. The addition of the luffa fruit powder did not negatively impact the participants’ liking of the couscous. However, it was associated with off-flavours, off-aromas, and an earthy flavour. The 40C and 60C samples were associated with the sensory attributes that increased consumer liking. The addition of the luffa fruit powder did increase the moistness perception of the couscous. The drying temperature influenced the flavour properties, as well as the phenolic content of the powder. This study identified the consumer perception of luffa fruit powder and introduced a new and novel ingredient, luffa fruit powder. This study was limited to the consumer perception of luffa fruit powder and identified the perception of consumers who do not regularly eat luffa fruit. This was a preliminary study, and future studies should continue to explore the use of luffa fruit powder in different foods and matrices. Future studies should investigate the sensory properties of the luffa fruit powder using the identified attributes by the consumers from this study to fully characterize them. Furthermore, studies should characterize the luffa fruit powder using different objective measurements (e.g., volatile organic compounds using gas chromatography) and investigate new processing methods and possible nutritional benefits.

## Figures and Tables

**Table 1:** Proximate composition (g/100 g) of the luffa fruit (conducted in triplicate, total phenolic content was conducted in duplicate).

Formulation	Protein	Lipid	Ash	Total Dietary Fibre	Moisture Content	Total Phenolic Content (mg/kg GAE)
40C	1.4 a ^1^ (0.4)	0.6 a (0.3)	5.2 a (0.5)	11.2 a (1.5)	12.0 a (0.5)	2968.89 a (22.1)
50C	1.6 a (0.3)	0.7 a (0.4)	5.5 a (0.5)	10.2 a (1.0)	3.6 b (0.3)	3750.9 b (104.6)
60C	1.8 a (0.2)	1.1 a (0.4)	5.0 a (0.4)	11.4 a (1.2)	1.8 c (0.5)	4895.2 c (148.1)

^1^ Means (standard deviation in brackets) in the same column, with the same letter, are not significantly different (95% confidence interval).

**Table 2:** The mean liking scores of the couscous samples with standard deviation in brackets.

Sample	Appearance	Aroma	Taste	Texture	Overall
Control	6.4 a ^1,2,3^ (1.6)	4.7 a (1.6)	5.4 a (1.3)	6.0 a (1.6)	5.4 a (1.4)
40C	5.7 b (1.7)	4.5 a (1.7)	5.0 a (1.3)	5.8 a (1.3)	5.1 a (1.5)
50C	5.8 b (1.6)	4.4 a (1.6)	5.3 a (1.2)	5.8 a (1.6)	5.4 a (1.6)
60C	5.7 b (1.6)	4.2 a (1.4)	5.1 a (1.1)	5.5 a (1.4)	5.1 a (1.3)

^1^ n = 88. ^2^ Results collected on a nine-point hedonic scale (1 = Dislike Extremely, 9 = Like Extremely). ^3^ Means, in the same column with the same letter, are not significantly different (95% confidence interval).

**Table 3:** The main concepts identified by the participants (n = 88) about the luffa fruit powder (the frequency of mention is included in brackets).

Concept (Frequency of Mention)	Examples
Mild Flavour (24%)	It doesn’t appear to have much flavour; Pretty benign; Needs something to liven it up; Tasted neutral; Not much flavour; Too bland; Mild; Subtle flavour
Healthy? (22%)	If it is high in nutrients, it might be a good way to incorporate some healthy bits for picky eaters!; Curious about its health benefits; Tastes healthy; If it adds nutrients, great!
Texture (20%)	Additional moistness; Adds moistness; Makes the couscous less dry; Pleasant mouthfeel
Earthy (19%)	The earthy taste was sometimes too strong; Earthy and woody; Very earthy; Too earthy and nutty
Off-Flavour (18%)	A bit of a different flavour; Needs to mask its flavour; Off flavour; Has a different taste to it; weird flavour
Off-aroma (12%)	Smells really bad; Smell is stronger than taste; Very strong scent; Weird smell; Unappealing odour
Positive (21%)	Love it!; I think it is good. I really liked it.; Would definitely try again; I like the texture; Great
Negative (20%)	Don’t think I would buy it; Not a fan; Did not like it; Not to my liking; I really don’t like it at all

## Data Availability

The original contributions presented in the study are included in the article, further inquiries can be directed to the corresponding author.
